# Airway nitrite is increased in extremely preterm infants with bronchopulmonary dysplasia

**DOI:** 10.1186/s12931-020-01508-8

**Published:** 2020-09-21

**Authors:** Samuel J. Gentle, Amelia Freeman, Rakesh P. Patel, Namasivayam Ambalavanan, Charitharth V. Lal

**Affiliations:** 1 Division of Neonatology, Department of Pediatrics, University of Alabama at Birmingham, 1700 6th Ave S, Birmingham, al 35233 USA; 2 Department of Pathology and Center for Free Radical Biology, University of Alabama at Birmingham, Birmingham, USA

**Keywords:** Bronchopulmonary dysplasia, Preterm infants, Nitrite, Nitric oxide

## Abstract

**Rationale:**

Bronchopulmonary dysplasia (BPD) is the most common complication of prematurity and significantly contributes to mortality and morbidity with few predictive biomarkers. Given that nitrites have been implicated in pathways associated with lung disease, we hypothesized that nitrite levels would be altered in the airways of premature infants diagnosed with BPD.

**Methods:**

This was a prospective cohort study of extremely low birth infants (< 28 weeks’ gestation) at the University of Alabama at Birmingham. Nitrite levels from tracheal aspirates (TAs) were compared between intubated and ventilated infants with BPD and gestation matched full term (FT) controls. TA derived nitrite levels from day one after birth were also compared between preterm infants who did and did not develop BPD.

**Results:**

Infants with BPD were found to have significantly elevated nitrite levels in their tracheal aspirates compared to gestation matched FT controls (*p* < 0.05). There was a trend for increased nitrite levels on postnatal day one in infants that developed BPD compared to infants that did not develop BPD (*p* = 0.05).

**Conclusions:**

In conclusion, nitrite levels are significantly increased in airways of infants with BPD. Data from a larger cohort are needed to further support the utility of nitrite for BPD prediction.

**Trial registration:**

Not applicable.

## Introduction

Bronchopulmonary dysplasia (BPD) is a common morbidity of preterm infants for which few biomarkers exist. Development of BPD results from a multitude of factors including inflammation, hyperoxia, disrupted pulmonary angiogenesis, and microbial dysbiosis. In addition to a paucity of biomarkers that predict disease, few therapies prevent disease development. Nitric oxide (NO), as well as precursors to NO production, have been evaluated as potential markers of disease and therapies for prevention.

Nitric oxide a signaling molecule produced throughout the airway, has many physiologic roles in the lung including vasodilatation and anti-inflammation. Moreover, in addition to other angiogenic growth factors, NO influences both pre- and postnatal pathways critical to lung development including airway branching morphogenesis and alveolarization [1, 2]. Nitric oxide production from L-arginine is catalyzed by nitric oxide synthase isoforms including neuronal, inducible, and endothelial nitric oxide synthase. In parallel, NO can be produced via reduction of nitrite, the source of which is either dietary intake, reported to be low in all infant dietary sources [3], or NO oxidation [4]. Given its significantly longer half-life, nitrite serves as a reservoir for subsequent NO production. Furthermore, nitrite deficiency has previously been associated with other lung diseases including pulmonary hypertension for which nitrites have been evaluated as a therapy for this disease [5].

Bronchopulmonary dysplasia in preterm infants is associated with pulmonary vascular remodeling and pulmonary hypertension, which in turn contributes to morbidity and mortality. Given the physiologic and developmental roles of NO which may ameliorate the contributory factors of inflammation and pulmonary vascular maldevelopment to BPD pathogenesis, NO has been extensively studied as a potential therapy for BPD prevention. In animal models inhaled NO improved gas exchange and angiogenesis [6], however, multiple randomized controlled trials of inhaled NO in preterm infants have not reduced death or BPD [7]. Whether nitrite measurement may predict BPD development or inhaled nitrite formulations can prevent BPD remains to be evaluated. We hypothesized that nitrite measured from tracheal aspirates (TA) would differ between extremely preterm infants that developed BPD compared to full term controls. In addition, we hypothesized that nitrite levels would differ between extremely preterm infants that did and did not develop BPD.

## Methods

We conducted a prospective cohort study at the University of Alabama Regional Neonatal Intensive Care Unit between 2015 and 2017. TAs were collected from three different patient populations: inborn or outborn extremely preterm infants (< 28 weeks’ gestational age) at birth or within 6 h of birth before surfactant administration, infants with BPD that were 36 weeks’ PMA and remained intubated, and gestational age matched full term controls. Samples taken at birth were compared between infants that did or did not develop BPD based on clinical outcome of severe BPD at 36 weeks’ PMA. Samples from infants 36 weeks’ PMA with BPD were compared to samples from gestational age matched full term infants that served as best available controls and were collected within 6 h after birth in infants intubated secondary to non-respiratory clinical indication (e.g. abdominal wall defect or perinatal depression). Institutional Review Board granted waiver of consent given that TA samples were collected from routine care. Demographic and clinical characteristics were collected from enrolled patients using the electronic medical record. The physiologic definition of BPD [8] at 36 weeks’ postmenstrual age was used.

TA samples were collected as per unit protocol, as described in our previous publications [9]. Prior to sample processing, samples were centrifuged and stored at -80 °C. For clinical data central tendency was reported as mean with standard deviation or median and interquartile ranges based on tests of normality. Nitrite levels were measured in the supernatant of TAs by triiodide based chemiluminescence on a Sievers NO analyzer and comparison to standard curves as previously described [10]. No infants were exposed to inhaled NO in the 7 days prior to TA collection. Test for normality performed on all continuous variables to inform appropriate statistical analysis. Demographic characteristics between preterm infants that did and did not develop BPD were compared using Fisher’s test for categorical variables, Mann Whitney test for continuous skewed variables, and unpaired t-test for continuous normally distributed variables. Nitrite levels taken from TAs were compared using a Mann Whitney test.

## Results

**Fig. 1:**
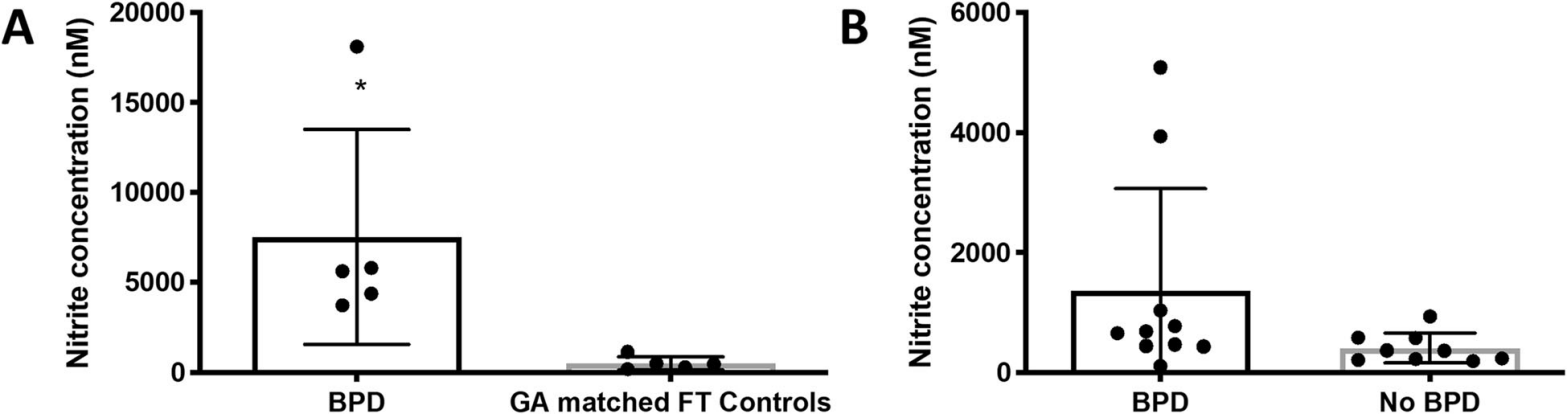
**a**, Nitrite concentration from tracheal aspirates in infants with BPD compared to gestational age matched full term controls. **b**, Nitrite concentration from tracheal aspirates at birth in infants that developed BPD compared to infants that did not develop BPD

Samples were taken at birth from 20 extremely preterm infants, at 36 weeks’ PMA in five infants with BPD, and at birth in five full term gestational age matched controls. In samples taken at birth, infants that developed BPD had a lower gestational age (23.7 vs 26.3 weeks) and weight (515 g vs 780 g). Full term infants were 37 weeks’ gestation at birth with a median birth weight of 3330 g. Additional characteristics for these infants are reported in Table 1. Nitrite levels in preterm infants with BPD were significantly higher than nitrite levels taken from full term matched controls (*p* < 0.05, Fig. 1a). There was a trend for higher nitrite levels at birth in infants that did develop BPD compared to infants that did not develop BPD (*p* = 0.05, Fig. 1b).

**Table 1:** Demographic and Clinical Characteristics of Enrolled Patients

	BPD (***n*** = 10)	No BPD (***n*** = 10)	***P*** value
Gestational age, weeks average (SD)	23.7 ± 2	26.3 ± 2	0.005
Birth weight median (IQ1, IQ3)	515 (480,615)	780 (693,988)	0.005
Male sex n (%)	1 (10)	7 (70)	0.02
White race n (%)	3 (30)	4 (40)	> 0.99
Histologic chorioamnionitis n (%)	4 (40)	3 (30)	> 0.99
Pre-eclampsia n (%)	5 (50)	5 (50)	> 0.99
Antenatal corticosteroids n (%)	9 (90)	10 (100)	> 0.99
Ventilator days average (SD)	53 ± 39	7 ± 4	< 0.002
Patent ductus arteriosus n (%)	1 (10)	2 (10)	> 0.99
Postnatal steroid exposure n (%)	9 (90)	0 (0)	< 0.001
Necrotizing enterocolitis stage ≥2 n (%)	3 (30)	1 (10)	0.58
Sepsis n (%)	4 (40)	1 (10)	0.30
Severe retinopathy of prematurity n (%)	2 (20)	1 (10)	> 0.99
Severe intracranial hemorrhage n (%)	3 (30)	1 (10)	0.58

*n* Number; *SD* Standard deviation; *IQ* Interquartile

## Discussion

Parallel NO production has emerged as a pathway critical to cardiopulmonary homeostasis through nitrite induced pulmonary vasodilation under hypoxic stress, protection from ischemia reperfusion damage, and regulation of vascular function [11]. Studies have implicated deficient NO bioactivity in pulmonary disease. Patients with pulmonary arterial hypertension had lower amounts of NO reaction products compared to healthy controls in samples from bronchoalveolar lavage; however, nitrite levels did not significantly differ [12]. It is unclear whether lower NO metabolites contribute to the pathogenesis of pulmonary hypertension or only provide a marker for disease severity by reflecting endothelial dysfunction, especially in preterm infants.

In the present study, infants with BPD had higher airway nitrite when compared to gestational-age matched controls. This suggests that NO synthesis and/or potentially NO that is biologically available from nitrite reduction is increased in the airways of these infants, which could result from airway exposure to hyperoxia and NO production by airway epithelia or leukocytes. Peroxynitrite, known to cause lung tissue injury, forms from superoxide radicals and NO and may subsequently decompose and increase nitrite in the preterm airway. Previous observational studies have reported elevated 3-nitrotyrosine levels and a trend for elevated nitrite levels in infants that developed BPD suggesting that markers of NO metabolism and peroxynitrite formation may serve as predictors for BPD in preterm infants [13, 14]. In addition, infants with BPD have also been reported to have higher urinary nitrite at 36 weeks’ PMA compared to infants with no BPD [15].

Nitrite supplementation may also have therapeutic benefit. In wild type mice exposed to prolonged hypoxemia, both nitrate and nitrite supplementation prevented pulmonary vascular remodeling and improved pulmonary hypertension [16]. Ethyl nitrite has previously been trialed in newborn infants with persistent pulmonary hypertension of the newborn wherein exposed infants (*n* = 7) had improvements in oxygenation and a reduction in the oxygen index [17]. If increased airway nitrite in BPD infants is compensatory and attenuates pulmonary vascular resistance, further nitrite supplementation may have therapeutic benefit. Conversely, nitrite may only have prognostic utility without protective effect, which may be further evaluated in animal models of BPD.

Due to the small sample size of the current study, it remains unclear whether airway nitrite levels at birth could serve as a predictive biomarker for BPD. Comparing TA nitrite at additional postnatal time points in infants that do and do not develop BPD may also warrant investigation. Given prior studies suggesting NO bioavailability may both predict and treat lung disease, further study of a larger cohort is needed.

## Abbreviations

BPD: Bronchopulmonary dysplasia
TA: Tracheal aspirate
NO: Nitric oxide
